# 一个遗传性低异常纤维蛋白原血症家系及其致病机制研究

**DOI:** 10.3760/cma.j.cn121090-20241011-00389-1

**Published:** 2025-06

**Authors:** 嘉伟 郑, 晓梅 卢, 李霞 郝, 林娜 卢, 嘉 杨, 丽东 赵, 栋彦 付, 端阳 王, 刚 王, 林花 杨

**Affiliations:** 山西医科大学第二医院血液科，山西医科大学出凝血疾病及恶性血液病研究中心，太原 030001 Department of Hematology, The Second Hospital of Shanxi Medical University; Research Center for Hemostatic Disorders and Hematologic Malignancies, Shanxi Medical University, Taiyuan 030001, China

## Abstract

先证者为32岁女性，因妊娠9月余、胎动减少和下腹部不适就诊。先证者及其父亲活化部分凝血活酶时间（APTT）、凝血酶原时间（PT）正常，纤维蛋白原活性和抗原水平降低，凝血酶时间（TT）延长，其母亲凝血指标检测结果均正常。超声检查示“左小腿肌间静脉血栓”。提取先证者及其父母外周血DNA，Sanger测序法检测FGA、FGB和FGG基因碱基序列，先证者及父亲均检出FGG基因6号外显子c.615A>C（p.γLeu205Phe）和8号外显子c.1121A>C（p.γTyr374Ser）杂合错义突变，生物信息学分析提示这两种基因突变可能是该遗传性低异常纤维蛋白原血症家系的致病机制。

## Abstract (en)

The proband was a 32-year-old female patient who sought medical attention for over 9 months of pregnancy, reduced fetal movement, and discomfort in the lower abdomen. The proband and her father had normal activated partial thromboplastin time and prothrombin time, decreased fibrinogen activity and antigen levels, and prolonged thrombin time, whereas the test results of her mother were normal. Ultrasonography showed intermuscular vein thrombosis in the left calf of the proband. Peripheral blood DNA was extracted from the proband and her parents, and Sanger sequencing was performed to detect the base sequences of the FGA, FGB, and FGG genes. The proband and her father had heterozygous missense mutations in exon 6 c.615A > C（p. Leu205Phe）and exon 8 c.1121A > C（p. Tyr374Ser）of the FGG gene. Bioinformatics analysis suggested that the two gene mutations may be the pathogenic mechanism of this congenital hypodysfibrinogenemia family.

遗传性纤维蛋白原缺陷症（congenital fibrinogen deficiency, CFD）是由纤维蛋白原基因FGA、FGB、FGG缺陷导致纤维蛋白原含量/结构异常的一种遗传性疾病，可分为无纤维蛋白原血症、低纤维蛋白原血症、异常纤维蛋白原血症和低异常纤维蛋白原血症四种亚型[1]。我们在一个遗传性低异常纤维蛋白原血症家系中发现FGG基因6号外显子c.615A>C（p.γLeu205Phe）和8号外显子c.1121A>C（p.γTyr374Ser）杂合错义突变，生物信息学分析初步表明两种基因突变具有致病性，可能是该家系的致病机制。

## 对象与方法

一、病例资料

患者，女，32岁，妊娠9月余，因为胎动减少和下腹部不适就诊。既往身体健康，无出血和血栓病史，无肝肾功能疾病。父母非近亲结婚，身体健康，无出血或血栓病史。查体：心肺腹无特殊阳性体征，皮肤黏膜及四肢无出血倾向，妊娠腹型，腹围117 cm，宫底高度31 cm，宫缩无，胎方位左枕前，未入盆，头先露，胎心145次/min。实验室及辅助检查：活化部分凝血活酶时间（APTT）32.2 s（参考值24.0～36.0 s），凝血酶原时间（PT）13.4 s（参考值11.0～14.5 s），凝血酶时间（TT）28.7 s（参考值10.2～20.1 s），Clauss法纤维蛋白原活性0.81 g/L（参考值2.00～4.00 g/L），其余凝血指标均正常。超声检查：左小腿肌间静脉血栓。本研究经山西医科大学第二医院伦理委员会批准（2023YX第159号）。受试者均签署知情同意书。

二、DNA测序

常规采集患者及家系成员血液标本，DNA试剂盒（美国Omega Bio Tek公司产品）提取DNA，Sanger测序法检测FGA、FGB和FGG基因外显子。

三、生物信息学分析

采用Clustal X-2.1-win将人类与NCBI公布的其他同源生物进行氨基酸保守性分析。用生物信息学网站对该突变可能产生的影响进行预测。从NCBI下载纤维蛋白原氨基酸序列，使用Swiss Model website（https://swissmodel.expasy.org/）网站建立蛋白质模型并使用Swiss Pdb Viewer4.10软件对突变进行分析。

四、纤维蛋白原的纯化、检测和凝固率测定

采用饱和硫酸铵法提纯纤维蛋白原[2]，−20 °C保存。聚丙烯酰氨凝胶电泳检测蛋白表达。准备10％分离胶和5％浓缩胶，60 V恒压45 min，90 V恒压2 h，考马斯亮蓝染色，蒸馏水洗涤，最后观察条带并拍照。

将纯化的纤维蛋白原（终浓度0.5 mg/ml）、NaCl（终浓度0.1 mol/L）、CaCl_2_（终浓度0.1 mmol/L）和凝血酶（终浓度1.5 U/ml，英国Abcam公司产品）快速混匀，分光光度计测量280 nm吸光度值（*A*_280初_）。37 °C孵育3 h，室温放置过夜后再次测量*A*_280_（*A*_280末_），根据下列公式计算纤维蛋白凝固率。

$$凝固率（％）=\frac{A_{280初}-A_{280末}}{A_{280初}}\times \text{1}00％$$

五、纤维蛋白聚集和溶解实验

将纯化纤维蛋白原和NaCl、CaCl_2_、凝血酶（终浓度同上）混匀，用酶标仪每隔30 s测量1次350 nm吸光度值，持续30 min。纯化的纤维蛋白原（终浓度0.5 mg/ml）和NaCl（终浓度0.12 mol/L）、CaCl_2_（终浓度0.32 mmol/L）、凝血酶（终浓度0.75 U/ml）、重组人组织纤溶酶原激活剂（tPA，终浓度0.075 µg/ml）、纤溶酶原（Plg，终浓度0.05 U/ml）混匀，用酶标仪每隔30 s测量1次350 nm吸光度值，持续10 min。根据测量数值，用GraphPad Prism 9软件绘制曲线。

## 结果

一、凝血系列检查结果

先证者及其父亲APTT、PT正常，纤维蛋白原活性和抗原水平降低，TT延长。其母亲的检测结果均正常（表1）。

**表1:** 遗传性低异常纤维蛋白原血症家系成员凝血指标检测结果

家系成员	APTT（s）	PT（s）	TT（s）	FIB∶C（g/L）	FIB∶Ag（g/L）	FIB∶C / FIB∶Ag比值
先证者	32.2	13.4	28.7	0.81	1.69	0.48
父亲	32.0	13.5	24.0	1.09	1.72	0.63
母亲	27.3	11.3	16.9	2.56	3.11	0.82
参考值	24.0～36.0	11.0～14.5	10.2～20.1	2.00～4.00	2.00～4.00	>0.70

**注** APTT：活化部分凝血活酶时间；PT：凝血酶原时间；TT：凝血酶时间；FIB∶C：纤维蛋白原活性；FIB∶Ag：纤维蛋白原抗原

二、基因测序结果

先证者及其父亲均检出FGG基因6号外显子c.615A>C（p.γLeu205Phe）和8号外显子c.1121A>C（p.γTyr374Ser）两个杂合错义突变（图1），而其母亲没有发现类似突变，因此这2个突变都来自于父亲（即顺式突变）。查询NCBI（https://www.ncbi.nlm.nih.gov/）、HGMD（https://www.hgmd.cf.ac.uk/ac/index.php）、Genome aggregation database（http://gnomad-sg.org/）和Fibrinogen database（https://site.geht.org/）等国际数据库，这2个突变均未见报道。

**图1:**
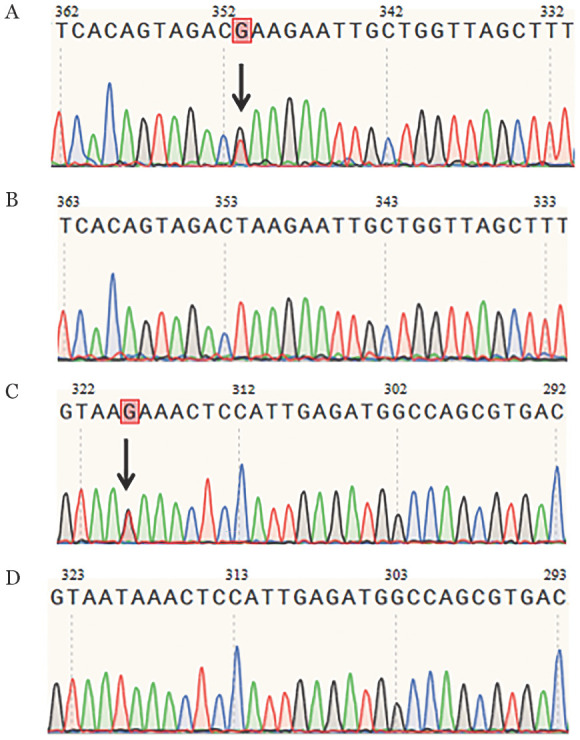
遗传性低异常纤维蛋白原血症家系FGG基因外显子测序结果 **A、B** FGG基因6号外显子c.615A>C（p.γLeu205Phe）突变及野生型；**C、D** FGG基因8号外显子c.1121A>C（p.γTyr374Ser）突变及野生型

三、突变纤维蛋白原的生物信息学分析

c.615A>C（p.γLeu205Phe）和c.1121A>C（p.γTyr374Ser）在其他同源物种间具有高度保守性，说明突变可能会产生不利影响。生物信息功能预测网站对新突变是否会产生不利影响进行进一步预测，Mutation Taster、Polyphen-2、Provean和SIFT对c.615A>C（p.γLeu205Phe）突变的预测结果分别为“致病”、“致病”、“中性”和“致病”，得分分别为0.999、1.000、−1.990、0.017；对c.1121A>C（p.γTyr374Ser）突变的预测结果均为“致病”，得分分别为0.999、1.000、−7.220、0.000。

四、突变纤维蛋白原的蛋白质模型分析

对于p.γLeu205Phe突变分析，蛋白质结构中亮氨酸是非极性疏水氨基酸和支链氨基酸，而突变后的苯丙氨酸是非极性疏水氨基酸和芳香族氨基酸，只改变了氨基酸，并没有氢键的改变（图2A、B）。对于p.γTyr374Ser分析，突变前酪氨酸属于芳香族氨基酸并与Leu224形成氢键，当发生突变后，氨基酸和氢键发生了变化，Tyr374突变为Ser374，并且和Arg223形成了新的氢键（图2C、D）。

**图2:**
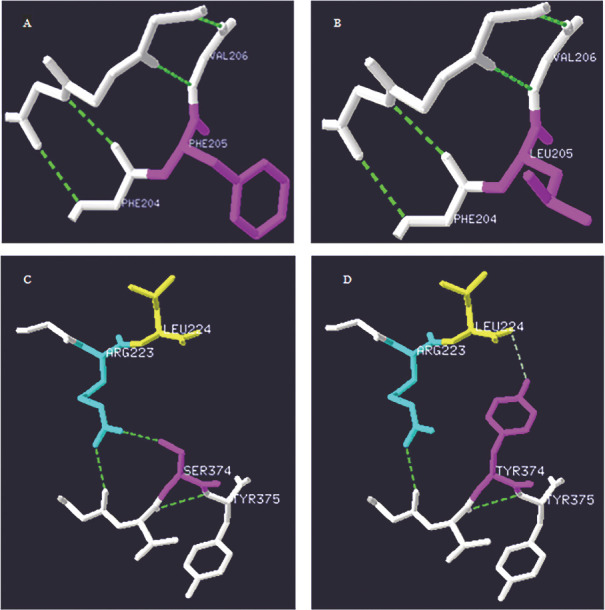
突变纤维蛋白原的蛋白质模型分析（粉色代表突变的氨基酸位置，绿色虚线代表氢键） **A、B** FGG基因6号外显子c.615A>C（p.γLeu205Phe）突变及其野生型；**C、D** FGG基因8号外显子c.1121A>C（p.γTyr374Ser）突变及其野生型

五、纤维蛋白原检测和凝固率实验

在SDS-PAGE凝胶结果中，未发现有异常肽链的产生（图3）。对照组、先证者、其父亲和母亲纤维蛋白原凝固率分别为（89.6±1.2）％、（71.2±5.1）％、（84.5±5.2）％、（92.1±5.8）％。先证者和其父亲的凝固率降低并不明显，这种改变可能对机体的止血和血栓影响不大。

**图3:**
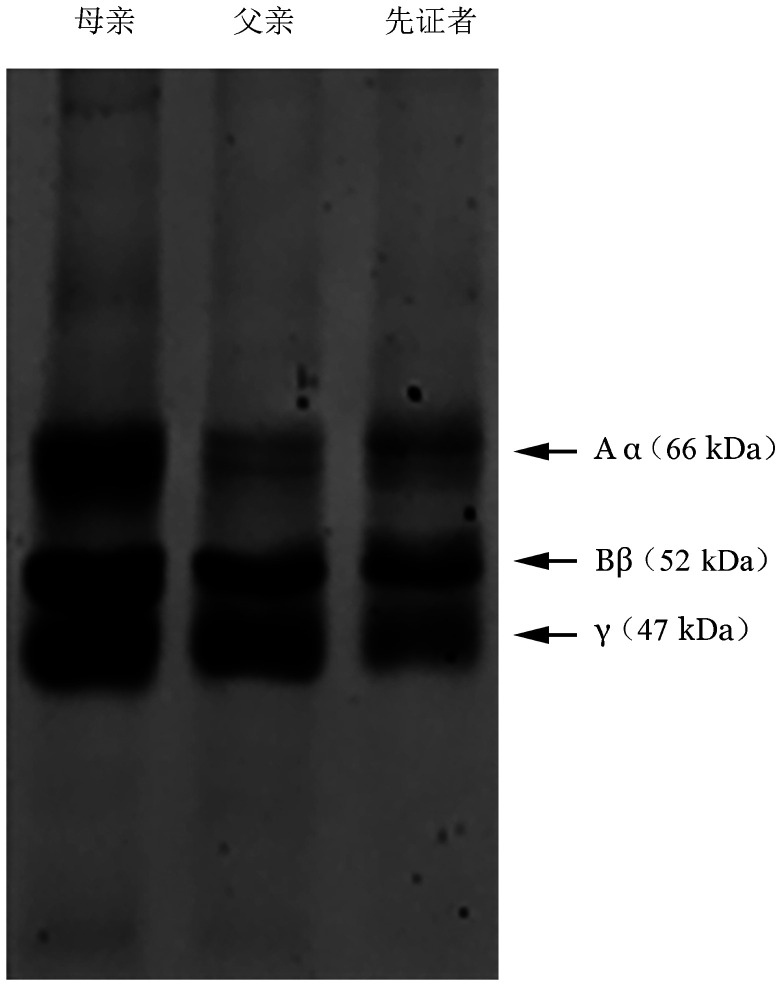
遗传性低异常纤维蛋白原血症家系成员纤维蛋白原凝胶电泳图

六、纤维蛋白功能实验

先证者的纤维蛋白最大聚集率和最大吸光度值（最大OD值）均低于正常对照组（图4A），纤维蛋白溶解曲线无明显差异（图4B）。

**图4:**
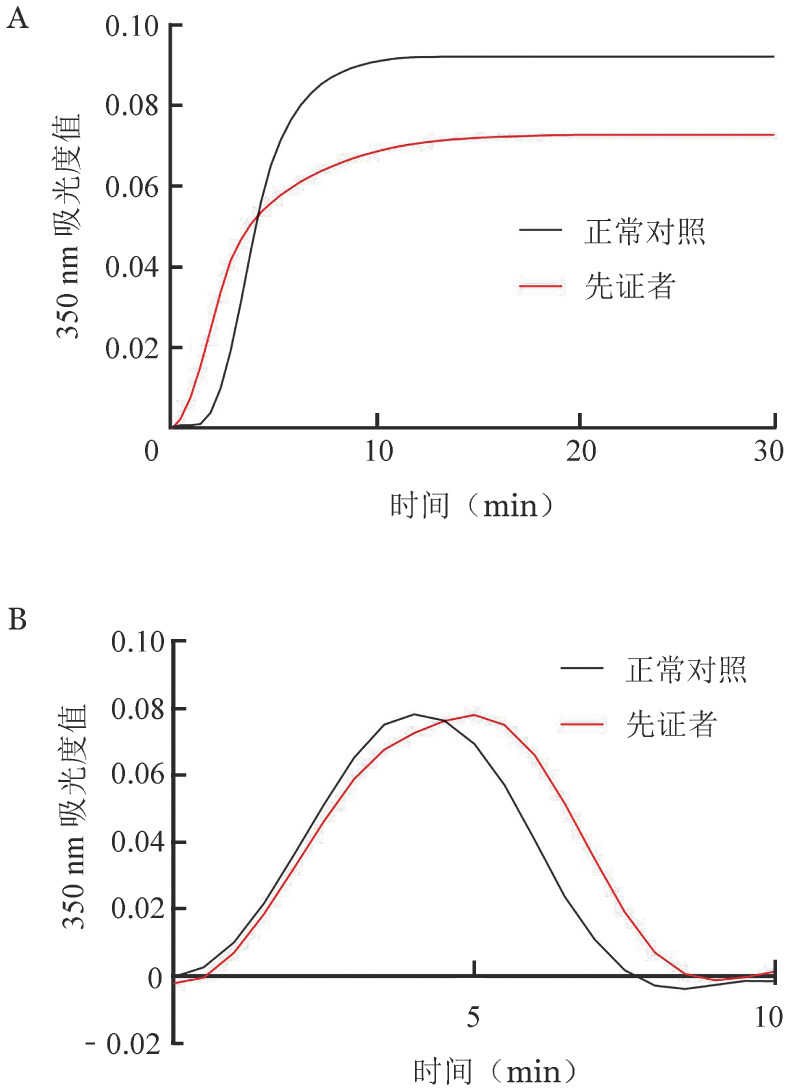
遗传性低异常纤维蛋白原血症先证者纤维蛋白聚集曲线（A）和纤维蛋白溶解曲线（B）

## 讨论

CFD发病率低，临床表现复杂但无特异性，诊断相对容易，但是鉴别诊断困难。本家系先证者纤维蛋白原活性和抗原水平均降低，根据ISTH分类标准[1]，属于遗传性低异常纤维蛋白原缺陷症。此前有研究发现，在纤维蛋白原结构异常的CFD患者中，其纤维蛋白原活性/抗原比值通常小于0.7，准确率为86％[3]，本家系先证者的纤维蛋白原活性/抗原比值为0.48。

CFD患者的临床表现差异大，包括从无症状、出血、血栓以及出血和血栓共存。本例患者为妊娠期女性，在妊娠检查中意外发现持续性纤维蛋白原降低，凝血系列检查显示患者及其父亲存在低纤维蛋白原和TT延长，基因测序检出FGG基因6号外显子c.615A>C（p.γLeu205Phe）及8号外显子c.1121A>C（p.γTyr374Ser）杂合突变。这两个突变是未见报道的新突变，存在于同一条染色体，均来自于先证者的父亲，属于顺式突变，是较为少见的一种基因突变。

在临床上，低纤维蛋白原水平合并血栓是较为少见的。先证者超声检查结果发现左小腿局部肌间静脉血栓。在后续的随访中，先证者在日常生活和整个妊娠期间并未出现其他部位的出血和血栓。先证者的父亲具有相同的突变，我们也对其父亲的既往病史进行了询问，但是其父亲既往身体健康，并没有明显的出血及血栓发生。

纤维蛋白功能实验表明突变导致纤维蛋白网络生成较慢，最后形成的纤维蛋白直径较细，使患者会有出血倾向。但是，本例患者却发生了小腿静脉血栓形成，可能与多方面的因素相关，因为妊娠期血液中其它的凝血因子含量也会升高，有可能促进血栓生成；随着妊娠期延长患者运动减少、卧床时间延长；虽然患者纤维蛋白原的活性和抗原水平降低，但是降低不明显。此前有动物研究也发现，小鼠的纤维蛋白原水平低于正常值的90％[4]，但是依旧能够起到止血作用，说明纤维蛋白原在一定范围的降低可能并不会使患者有明显的出血表现，但是综合其他因素可能会出现低纤维蛋白原水平合并血栓。临床上主要应该以预防血栓为主，特别是妊娠期，但是在一些特殊情况下，如手术、分娩等，应根据指南考虑预防性补充纤维蛋白原，预防出血。

在纤维蛋白溶解实验中，患者和对照病例并未显现明显的差异，说明低纤维蛋白原不是由纤维蛋白溶解亢进导致。p.γLeu205Phe和p.γTyr374Ser这两个新突变的位置都位于γ链的末端的球状区域内，突变可能会影响‘旋钮A和孔a’的结合，导致纤维蛋白单体之间的相互交联受损。此前有研究已经发现的与孔a相关的可能位点有γPhe295-Thr305、γTrp315-Trp330和Trp335-Asn365[5]。

p.γLeu205Phe是位于γ链球状区域B区（γGlu183-Asn325），生物信息学功能预测是有害的。Kotlín等[6]报道p.γPhe204Val突变，在该突变中纤维蛋白聚集和凝块裂解正常，但是扫描电镜下的纤维蛋白网络直径更宽。Zhou等[7]曾报道1名33岁妊娠女性，体检发现纤维蛋白原水平降低，基因测序结果为AαArg16His和γAsp185Asn复合突变，但患者并无出血及血栓史。Chinni等[8]报道的p.γGly191Val突变患者也没有特殊症状和体征。p.γTyr374Ser生物信息学预测也是有害的。Simonis等[9]报道了1例身体健康的18岁女性，在口服避孕药后，逐渐出现呼吸困难伴纤维蛋白原水平持续低下、肺动脉和右心房血栓形成，基因测序检出p.γTyr374Cys杂合突变。Tyr374与Arg375相邻，此前有报道p.γArg375Trp杂合突变导致大量的纤维蛋白原蓄积在肝细胞的内质网中，引起长时间的肝功能异常[10]。Kotlín等[11]报道了Gly377Ser突变导致患者纤维蛋白原降低，通过凝血系列检查、纤维蛋白肽释放、纤维蛋白凝固率和聚集实验及扫描电镜观察，均未发现纤维蛋白原结构异常，但是患者在穿刺和手术后发生异常出血，可能是纤溶亢进引起。

综上，我们在一个遗传性低异常纤维蛋白原血症家系中发现了对纤维蛋白原功能和结构产生不利影响的FGG基因6号外显子c.615A>C（p.γLeu205Phe）和8号外显子c.1121A>C（p.γTyr374Ser）杂合错义突变，生物信息学分析提示这两种基因突变可能是该家系的致病机制。
